# Fabric-Based Triboelectric Nanogenerators

**DOI:** 10.34133/2019/1091632

**Published:** 2019-11-24

**Authors:** Jinmei Liu, Long Gu, Nuanyang Cui, Qi Xu, Yong Qin, Rusen Yang

**Affiliations:** ^1^School of Advanced Materials and Nanotechnology, Xidian University, Xi'an 710071, China; ^2^Institute of Nanoscience and Nanotechnology, Lanzhou University, Lanzhou 730000, China

## Abstract

In the past decades, the progress of wearable and portable electronics is quite rapid, but the power supply has been a great challenge for their practical applications. Wearable power sources, especially wearable energy-harvesting devices, provide some possible solutions for this challenge. Among various wearable energy harvesters, the high-performance fabric-based triboelectric nanogenerators (TENGs) are particularly significant. In this review paper, we first introduce the fundamentals of TENGs and their four basic working modes. Then, we will discuss the material synthesis, device design, and fabrication of fabric-based TENGs. Finally, we try to give some problems that need to be solved for the further development of TENGs.

## 1. Introduction

Portable electronics with multifunctionality and wearability develop rapidly and are a great benefit to communication, personal health care, and environmental monitoring. On the other hand, textiles have been used by mankind for a long time, and they are essential to daily life. They are bendable, rollable, portable, and foldable and can be adapted to our daily life environment. Thus, many research groups have explored the integration of electronic functionalities into common textiles for wearable electronics. They incorporated conductive nanostructures, metallic nanomaterials, hybrid nanocomposites, and polymer nanocomposites on the surface of flexible cotton materials [1–7] and developed transistors [8–11], light-emitting diodes [12–14], and sensors [15–23]. These wearable electronics can monitor and record human activities. However, they cannot operate without electrical energy, making the development of wearable energy-harvesting and storage devices essential [24–30].

There are many studies on wearable devices to harvest energy from a human body, such as piezoelectric nanogenerators [31–35], thermoelectric nanogenerators [36–40], and triboelectric nanogenerators [41–46]. The newly invented triboelectric nanogenerator (TENG) that is based on the triboelectrification effect and electrostatic induction is superior when it comes to converting low frequency mechanical energy into electric power [47, 48]. A TENG with an output current of 10 mA and an output voltage of 500 V was demonstrated [49]. The charge density reached 1020 *μ*C m^−2^ [50], the area power density reached 500 W m^−2^ [51], and the volume power density reached 15 MW m^−3^ [51]. An instantaneous conversion efficiency of ≈70% has been demonstrated [52]. The high output makes TENGs an ideal power source for common wearable electronics. In addition, with its prominent advantages like universal materials, simple structures, low cost, and easy fabrication, TENG has attracted considerable interest in wearable electronics [53–55]. Fabrics can be easily woven into a textile to manufacture a TENG; thus, fabric-based TENGs are suitable for integration with fabrics for wearable electronics [56–60].

Here, this paper provides an overview of the significant development in fabric-based TENGs. Their fundamental device design, experimental procedures, and device performance are discussed. Conclusions and perspectives are given at the last part. This review supplements ideas for researchers actively engaging in the field of wearable energy-harvesting technologies.

## 2. Invention and Working Principle of TENG

Triboelectrification is one of the most common phenomena observed in our everyday activities [61–63]. Friction between materials with different dielectric constants would lead to the occurrence of triboelectrification on contacting surfaces. The triboelectric nanogenerator (TENG) utilizes triboelectrification to convert mechanical energy into electricity. The first TENG was reported in 2012, in which a power density of 31.2 mW cm^−3^ and an output voltage of 110 V were obtained through the contact-separation of a polymethylmethacrylate (PMMA) layer and polyimide nanowires [47]. Up to now, TENGs based on various materials and structures have been developed for a wide range of applications such as ambient energy harvesting [64], internet of things [65], batteryless medical systems [66], and self-powered sensing techniques [67].

**Figure 1:**
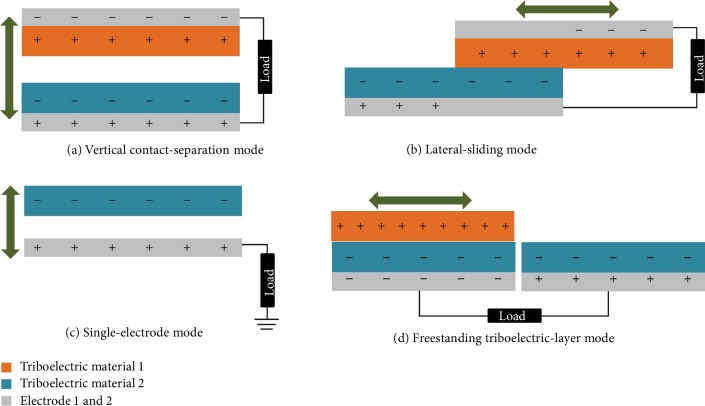
Four working modes of TENGs.

A TENG can convert mechanical energy into electricity by using the coupling effect of triboelectrification [68–70] and electrostatic induction [71–73]. Based on this working principle, four basic modes of TENGs have been proposed, including the vertical contact-separation mode, the lateral-sliding mode, the single-electrode mode, and the freestanding triboelectric-layer mode as shown in Figure 1. The contact-separation mode was first reported in 2012 [47]. The TENG works through the relative motion perpendicular to the material surface. The potential difference between two electrodes changes with the gap between material surfaces, which lead to a flow of external electrons. A lateral-sliding mode was reported in a TENG in 2013 [74]. The TENG uses the relative displacement in the direction parallel to the material surface and can be implemented in a compact package via rotation-induced sliding. A TENG working in a single-electrode mode also appeared in 2013 [75]. It takes the ground as the reference electrode and is versatile in harvesting energy from a freely moving object without attaching an electric conductor. Finally, the freestanding triboelectric-layer mode TENGs were proposed in 2014 [76]. This mode was developed upon the single-electrode mode. Instead of using the ground as the reference electrode, it uses a pair of symmetric electrodes, and electrical output is induced from asymmetric charge distribution as the freely moving object changes its position. One thing worth noting is that conjunction or hybridization of different modes may further improve the performance of TENGs.

## 3. Fabric-Based TENGs

Wearable TENGs placed on the human body should be comfortable and harmless for the wearer, and they should conveniently harvest energy from body movement. In the textile industry, fabric cloth is a flexible and porous material made from threads, fibers, and yarns. Now, many groups have made great efforts in developing fabric-based TENGs to harvest mechanical energy from human body motion.

### 3.1. Fabric-Based TENG with a Planar Structure

**Figure 2:**
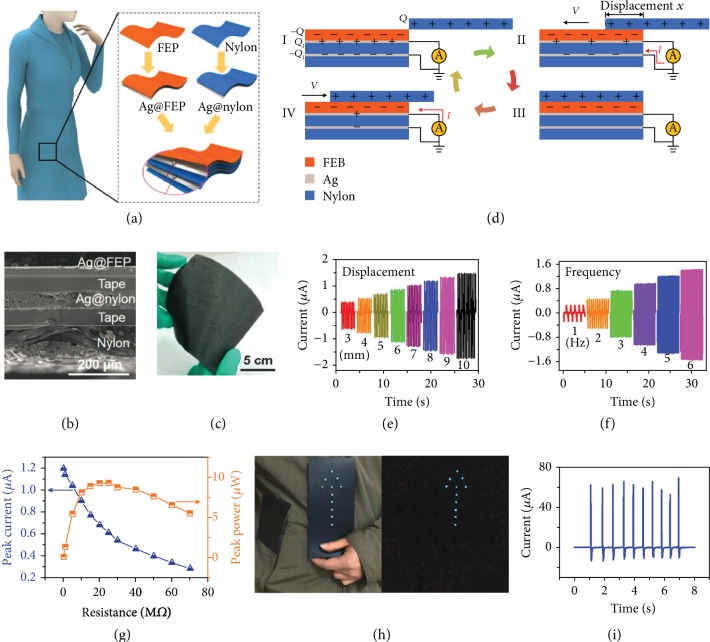
(a) Schematic diagram of the fabrication process of the power shirt. (b) Cross-sectional SEM image of the power shirt. (c) Digital picture of a 10 cm × 10 cm-area power shirt. (d) Working mechanism of the power shirt. (e) Short-circuit currents of the power shirt under a given sliding frequency of 3 Hz with different sliding displacements from 3 mm to 10 mm. (f) Short-circuit currents for the power shirt under a given sliding displacement of 5 mm with different sliding frequencies of 1 Hz to 6 Hz. (g) Peak output currents and peak power as a function of the external load resistances at a given sliding frequency of 5 Hz and displacement of 5 mm. (h) Power shirt for clothing ornament and risk warning. Digital pictures of LEDs powered by the power shirt and sleeve. (i) The output current for lighting up the LEDs.

In 2015, Li et al. reported a TENG-based power shirt to convert ambient mechanical energy into electric power as shown in Figure 2 [77]. A Ag layer was sputtered on fluorinated ethylene propylene (FEP) film and nylon clothes as two electrodes to form the Ag@FEP and Ag@nylon components. Then, the Ag side of the Ag@FEP component and the nylon side of the Ag@nylon component were adhered together by a double-sided adhesive tape. Another piece of nylon cloth was adhered to the Ag side of the Ag@nylon component as the protecting layer (Figures 2(a)–2(c)). The short-circuit current of the TENG with different sliding displacements under a given sliding frequency of 3 Hz is shown in Figure 2(e), in which the peak value of the output short-circuit current increases from 0.36 *μ*A to 1.48 *μ*A with the increase of sliding displacement from 3 mm to 10 mm. The short-circuit current of TENG driven at different sliding frequencies under a given sliding displacement of 5 mm is shown in Figure 2(f). The peak value of the output short-circuit currents increased from 0.24 *μ*A to 1.42 *μ*A as the sliding frequency changed from 1 Hz to 6 Hz, reaching a maximal short-circuit current density of 0.37 *μ*A cm^−2^. The peak power reached the maximum of 9.3 *μ*W when the external resistance was 25 MΩ, and a maximum peak power density of 4.65 *μ*W cm^−2^ was achieved (Figure 2(g)). To demonstrate its application, the TENG was slid with the sleeve and the peak currents of 70 *μ*A was generated, which lit up 11 blue LEDs, indicating its potential as a clothing ornament and risk warning (Figures 2(h) and 2(i)). This work develops a simple path for human body energy harvesting and promotes the development of wearable electronics.

### 3.2. Output Performance Improvement of Fabric-Based TENGs

#### 3.2.1. Performance Improvement with Surface Morphology

**Figure 3:**
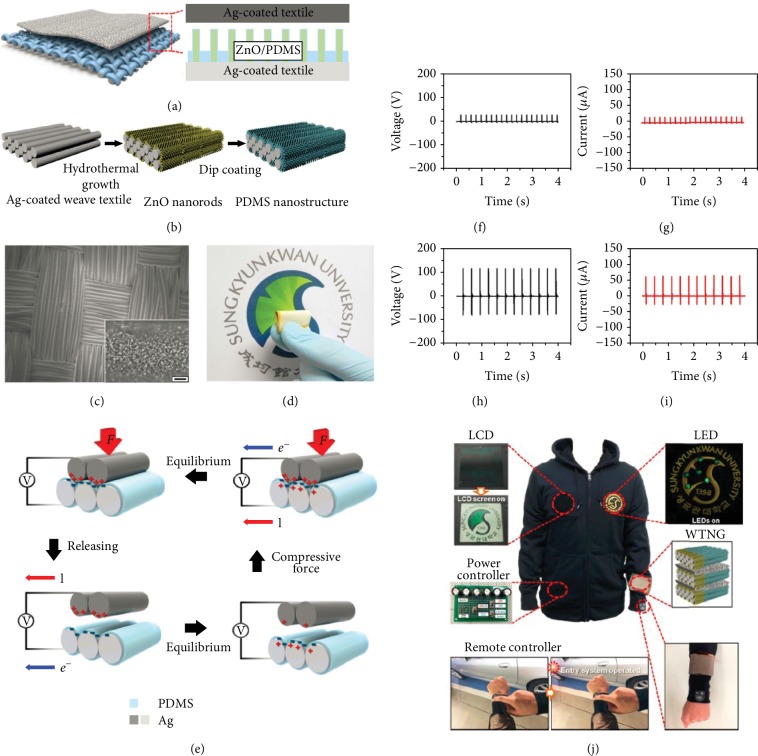
Schematic diagram (a) and fabrication process (b) of a nanopatterned fabric-based TENG. (c) SEM image of the bottom fabric with a nanopatterned PDMS. Inset shows a high-magnification SEM image that clearly shows ZnO NW-templated PDMS nanopatterns. (d) Photograph of a flexible and foldable nanopatterned fabric-based TENG. (e) The power-generating mechanism of the fabric-based TENG. The output voltage (f) and current (g) of fabric-based TENGs with a flat PDMS. The output voltage (h) and current (i) of fabric-based TENGs with a nanopatterned PDMS. (j) Self-powered jacket, including a commercial LCD, LEDs, and a remote control working by a nanopatterned fabric-based TENG.

To increase the output performance of a fabric-based TENG, the surface of the triboelectric material could be modified to increase the friction or contact area. Seung et al. demonstrated a fabric-based TENG with a nanopatterned surface [78]. As shown in Figures 3(a)–3(c), the nanopatterned fabric-based TENG consists of two layers. A Ag-coated fabric on the top is used as the electrode and positive triboelectric material, and a Ag-coated fabric of PDMS nanoparticles based on ZnO nanowire arrays at the bottom is used as a negative friction material. ZnO nanowires were grown on a Ag-coated fabric by a hydrothermal growth method, and then PDMS was coated on the grown ZnO nanowires by a dip-coating process.  Figure 3(d) is a photographic image of the fabric-based TENG. The power-generating mechanism of the fabric-based TENG is shown in Figure 3(e). The nanopattern can increase the friction and contact area between Ag and PDMS in a fabric of the same size, effectively creating many triboelectric charges on both surfaces. The fabric-based TENG with a nanopatterned PDMS produced higher output voltages and output currents of 120 V and 65 *μ*A compared to 30 V and 20 *μ*A for a flat PDMS without nanopatterns as shown in Figures 3(f)–3(i). A multilayer-stacked TENG was demonstrated to further enhance the total power output. At last, the fabric-based TENG was integrated into a jacket containing a LCD screen, LEDs, and a remote control for a keyless vehicle entry system. By harvesting mechanical energy generated when tapping a hand, the fabric-based TENG can turn on the LCD screen and drive six LEDs at the same time. Also, a commercial capacitor (1200 *μ*F) was charged by a fabric-based TENG as the power source for a remote control for keyless vehicle entry systems, demonstrating its applicability for self-powered smart suites (Figure 3(j)).

Another fabric-based TENG with an increased triboelectric output is developed by forming nanostructures on the surface of a textile platform using an assembly of a Au-coated fabric and polydimethylsiloxane (PDMS) [79]. To enhance the surface friction energy, thermally evaporated Al nanoparticles (NPs) were conjugated with Au-coated textile top electrodes. Using the rectified DC current signals obtained from the fabric-based TENG, a commercial capacitor was charged and a light-emitting diode (LED) was turned on. When attached to a human arm, an output voltage and current of 139 V and 39 *μ*A were generated when the TENG was bent and released, respectively. Its high efficiency can be attributed to its enhanced surface roughness induced by uniformly distributed Al NPs on the textile electrodes. In 2016, Guo et al. demonstrated a high-performance fabric-based TENG by using a nylon cloth as the substrate that was dip coated with Ag NWs, PDMS, and fluoroalkyl silane (FAS) in sequence [80]. The Ag NWs, PDMS, and FAS layer functioned as the electrode, the triboelectric material, and the chemically modified surface, respectively. The other electrode was composed of a commercial PET cloth, double-sided tape, and Al foil electrode. The addition of the FAS layer resulted in increased *V*_OC_ and *I*_SC_ from 53.2 V to 575 V and 1.1 *μ*A to 12.1 *μ*A compared to the device without the FAS layer. In the presence of FAS modification, the maximum output power density reached 2.8 W m^−2^. The TENG was used to charge commercial capacitors and light LEDs, showing its potential in powering wearable electronics.

#### 3.2.2. Performance Improvement with Structure Design

**Figure 4:**
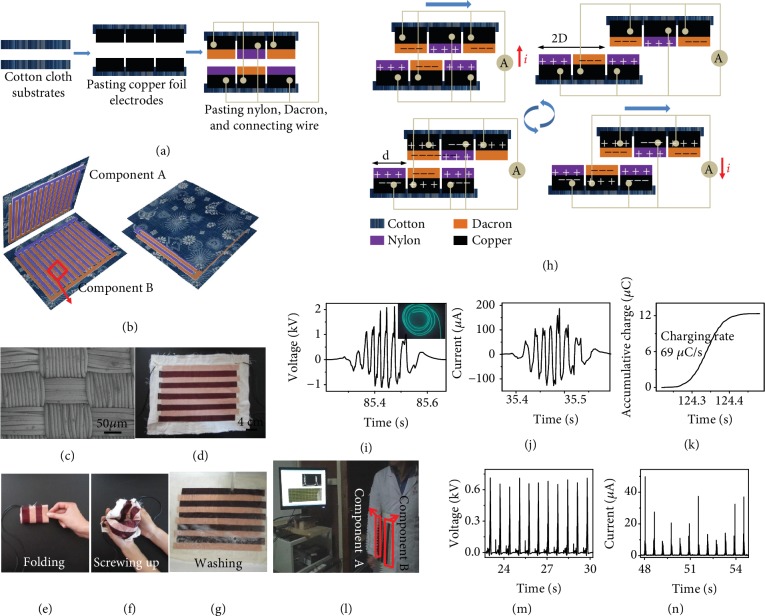
(a) Fabrication process and (b) schematic diagram of the cloth-based wearable triboelectric generator. (c) SEM image of the microstructure of a nylon cloth's surface. (d) Photograph of half of the part of the fabricated wearable triboelectric generator. (e–g) Photograph of the wearable triboelectric generator under folding and screwing up operation and washing process. (h) The working mechanism of the wearable triboelectric generator. (i) Enlarged view of the voltage output signal and (j) current output signal at an average sliding velocity of 1.7 m/s. Inset: an EL lamp lighted by the generator. (k) Charging curves of the triboelectric generator. Via a full-wave bridge, the electrons of one wave packet are pumped into a capacitor of 100 *μ*F. (l) Photograph of a working TENG sewn on clothes. (m, n) Rectified output voltage and current of TENG when it was attached to a swing arm.

Periodic lattice structures can be used to effectively improve the output performance of TENG. Our group has reported a fabric-based TENG composed of two layered structures with nylon cloth and Dacron cloth as triboelectric materials [81]. As shown in Figures 4(a)–4(d), two cotton cloths were used as substrates while ten strip electrodes of the same size were attached in a grating structure with a certain gap between electrodes. The nylon cloth strip and the Dacron cloth strip of the same size were alternately put on ten electrodes with a double-sided tape. The nylon-covered electrode and the Dacron-covered electrode were connected in parallel. The flexible performance and washability of the wearable triboelectric generator were tested, and their corresponding photographs are shown in Figures 4(e)–4(g). The lateral-sliding mode of the wearable triboelectric generator is demonstrated in Figure 4(h). When a relative displacement of 32 cm in one direction is applied under an average sliding speed of 1.7 m s^−1^ with an average pressure of 505 Pa, *V*_OC_ and *I*_SC_ of this fabric-based TENG can reach 2 kV and 0.2 mA, respectively. Its output in one motion has 16 peak signals and a 43 Hz frequency. It can be easily adjusted by controlling the sliding speed and the width of the grating unit (Figures 4(i) and 4(j)). Since the generated output has an AC-type power with high voltage and high frequency, it can be used as a power source for an electroluminescence (EL) lamp operating under these conditions. A tubular EL lamp with a length of 80 cm and a diameter of 2.5 mm was connected to the device and can emit a bright green light as shown in the inset of Figure 4(i). The AC-type output can be converted into DC-type output via a rectifying bridge, and the converted output can be stored in a capacitor. Using the output of this TENG in a single sliding motion in one direction, the charging rate reached 69 *μ*C s^−1^ and the charging rate per unit area of the device was 1.23 mC m^−2^ s^−1^ (Figure 4(k)). To assess the ability of this fabric-based TENG to harvest mechanical energy generated by human motion, two layers of the device have been attached to the waist and inner forearm of the cloth, so that two layers were in good contact when arms swing naturally (Figure 4(l)). The output from the swing of the arm was rectified. It had a voltage of 0.7 V and a current of 50 *μ*A (Figures 4(m) and 4(n)). This wearable TENG has shown a great potential to be developed into a power source for increasingly popular portable electronic devices and form a unique energy supply network.

**Figure 5:**
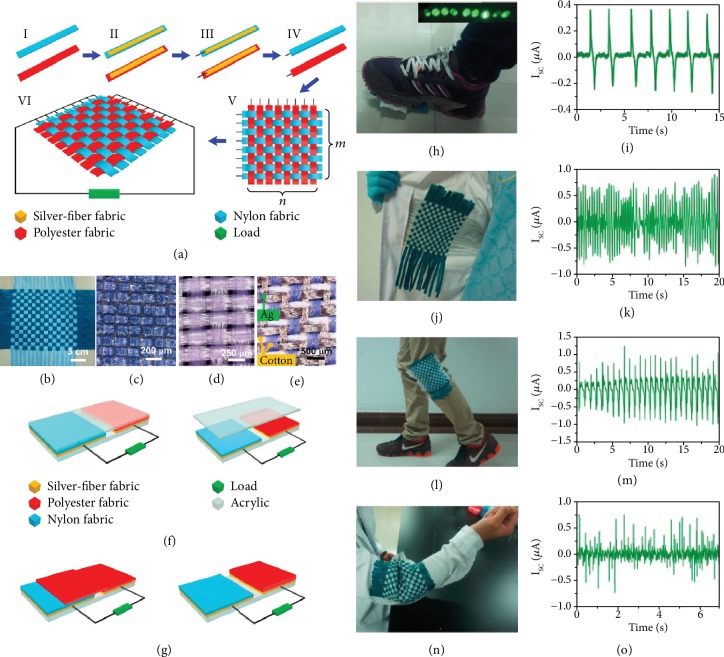
(a) Schematic diagram illustrating the fabricating process of a W-TENG. (b) Digital photography of a 16 × 16 W-TENG. Electron optical microscope images of the (c) nylon fabric, (d) polyester fabric, and (e) silver fiber fabric. (f) Structure and working process of the freestanding triboelectric-layer TENG, where acrylic works as the freestanding triboelectric layer in a vertical contact-separation mode. (g) Structure and working process of the freestanding triboelectric-layer TENG, where acrylic works as the freestanding triboelectric layer under a lateral-sliding mode. (h) A power-generating shoe (P-shoe) that could harvest energy from footsteps and light up about 9 LEDs. (i) The current generated by the P-shoe. (j) A W-TENG integrated in a coat harvesting energy from the shaking of clothes. (k) The current generated by the coat. (l) The W-TENG harvesting energy from bending of leg joints. (m) The current generated by the leg joint. (n) The W-TENG harvesting energy from arm joints. (o) The current generated by the arm joint.

A woven-structured TENG based on a freestanding mode was developed [82]. The TENG utilized contact-friction between fabrics or between a fabric and other objects by human motion as shown in Figure 5. This fabric-based woven TENG consisted of a homemade conductive Ag fiber fabric as the electrode material and commodity nylon fabric and polyester fabric as the positive and negative triboelectric materials, respectively. As shown in Figures 5(a)–5(e), the woven-structured TENG was fabricated with two types of fabric strips by a plain weaving method. The silver fiber fabric that served as the electrode was a mixed textile of Ag fibers and cotton fibers (Figure 5(e)). Its working mechanism is divided into a vertical contact mode using a freestanding acryl plate and a lateral-sliding mode using a polyester fabric as the freestanding triboelectric layer (Figures 5(f) and 5(g)). When attaching the TENG to a sole to harvest mechanical energy generated from the foot step, an output current of about 0.3 *μ*A was produced in one footstep and could be directly used as a power source for nine LEDs. The number of steps made by a person can be calculated based on output signals in one footstep (Figures 5(h) and 5(i)). When the TENG was applied inside a coat to harvest mechanical energy generated by shaking the clothes, an output current of about 0.75 *μ*A was produced (Figures 5(j) and 5(k)). A TENG was integrated into the knee parts of pants and the arm parts of a coat to harvest the mechanical energy generated by movements of the legs and arms, and output currents of about 0.9 *μ*A and 0.75 *μ*A were produced from human movements (Figures 5(l)–5(o)). Being flexible, washable, breathable, and wearable, the woven-structured TENG provides a new solution for the power supply of wearable devices.

Tian et al. proposed a TENG using a Ni-coated PE (conductive textile) and a silicone rubber-coated conductive textile as the woven substrate [83]. When palm skin was used as the freestanding triboelectric layer, the *I*_SC_ and *V*_OC_ of the single-layer and double-layer TENG were 60 *μ*A and 140 *μ*A and 500 V and 540 V, respectively. The maximum peak power of the double-layer TENG reached 22.3 mW at a load resistance of 10 MΩ, corresponding to a power density of 8,920 mW m^−2^. Ning et al. reported a washable textile-structured TENG based on the nanofibrous polytetrafluoroethylene (PTFE) polymer with high hydrophobicity [84]. A stained TENG can be easily and quickly cleaned by washing in water. The TENG can be sewed on clothes and effectively convert mechanical energy to electricity by the friction with the material of clothes. When operated by swinging arms while people are walking or running, an output voltage and current of 1050 V and 22 *μ*A were obtained, respectively. The generated electric energy could directly power a night running light and a digital watch without any energy-storage process.

From the above discussion, it can be found that the performance of the TENGs could be effectively improved through structure design. When TENGs were made with fabrics in a more elaborate structure, the triboelectric effect can be enhanced, and the surface's tribocharge density can be increased, which benefits the increase of the output current, output voltage, and peak power density. Furthermore, for the fabric-based TENGs working in the sliding mode, the output frequency of the electric signals can be increased. The output performance for fabric-based TENGs with different structures is shown in Table 1.

### 3.3. Environmental Adaptability Improvement of Fabric-Based TENGs

**Figure 6:**
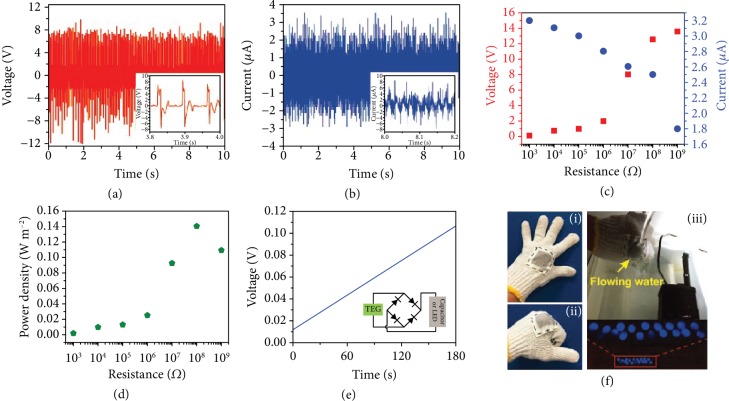
Output voltage (a) and output current (b) of PET fabric-based WTEG. The flowing rate of water was 6 mL s^−1^, and the distance between WTEG and the outlet of pipe was 10 cm. The insets are their magnified signals. (c, d) Dependence of output voltage, output current, and instantaneous power density of PET fabric-based WTEG on the resistance of external load. (e) Charging curve of 10 *μ*F capacitor by the PET fabric-based WTEG. (f) PET fabric-based WTEG was incorporated into a cotton glove (i and ii) for harvesting water energy to drive the commercial LEDs (iii). PET fabric-based WTEG with effective dimensions of 1.5 cm × 1.5 cm were used here for output tests; effective dimensions of 3 cm × 3 cm were operated here for driving LEDs.

Harvesting energy from water flow using triboelectric generators (TEGs) based on our daily wearable fabric or textile has practical significance. Xiong et al. reported a wearable all-fabric-based TEG for water energy harvesting with additional self-cleaning and antifouling properties [85]. In this work, a hydrophobic cellulose oleoyl ester nanoparticle- (HCOENP-) coated PET fabric was employed to fabricate the TEG. The HCOE was synthesized by grafting with the oleoyl chloride via esterification modification based on the microcrystalline cellulose (MCC) and transferred from a weak polar solvent to a polar solvent via nanoprecipitation to obtain the HCOENPs. Then, the ethanol suspension of HCOENPs was sprayed on the superhydrophilic PET fabric to construct a quasimonolayer hydrophobic coating. After having been sprayed by HCOENPs, the PET fabric showed a superhydrophobicity with a high static contact angle (SCA, 162.1°). The output voltage and current of the fabric-based TEG reached 15 V and 4 *μ*A, respectively (Figures 6(a) and 6(b)). An instantaneous output power density of 0.14 W m^−2^ at a load resistance of 100 MΩ was obtained (Figures 6(c) and 6(d)). To demonstrate the application, the PET fabric-based TEG was used to charge a 10 *μ*F capacitor to 2.7 V in 180 s after converting the AC output to DC output via a full-wave rectifying bridge (Figure 6(e)). It was then woven into the cotton glove and lit up commercial LEDs as exhibited in Figures 6(f)–6(h), showing its flexibility and deformability.

To work well under humidity conditions, Kim et al. demonstrated a humidity-resistant, fabric-based TENG by depositing self-assembled monolayers (SAM) to increase the hydrophobicity of the fabric surface [86]. The TENG was demonstrated as a wearable device by mounting it onto various parts of the human body, which could be used in self-powered smart clothes or wearable healthcare devices. In 2018, Kim et al. reported a simple method to fabricate a washable, breathable, and wearable TENG. The TENG harvested the energy of triboelectricity through an enhanced friction surface area that was made of a gold nanodot-pattern crafted by electron-beam sputtering on an inexpensive polyurethane surface [87]. The gold was deposited as regular small islands and then treated under oxygen plasma and etched into a nanodot-pattern on a polyurethane surface. Based on this structure, the TENG could convert mechanical energy into electrical energy with a maximum output of 2 *μ*W in a sliding mode. They designed a self-powered wearable device integrated with clothes to harvest different kinds of mechanical energies from human motion. A waterproof fiber was fabricated with a flutter membrane. Triboelectric charges as a function of the airflow speed was measured. At a mild wind speed, the fabricated TENG showed a maximum output of 70 *μ*W. Recently, Lai et al. presented a waterproof and fabric-based multifunctional triboelectric nanogenerator (WPF-MTENG), which produced electricity from both natural tiny impacts (rain and wind) and body movements. It can not only serve as a flexible, adaptive, wearable, and universal energy collector but also act as a self-powered, active, fabric-based sensor [88]. A WPF-MTENG-based keypad as a self-powered human-system interface is demonstrated on a garment for remotely controlling a music-player system, presenting a promising approaching for alternative energy.

### 3.4. Integration of Fabric-Based TENG

To power portable electronics, wearable energy-harvesting and storage devices are essential. Thus, a great deal of fabric-based TENGs were developed to be integrated with an energy storing device. Pu et al. reported a fabric-based TENG fabricated by plain-weaving strips of Ni-coated PE fabric (Ni-cloth) and parylene-coated Ni fabric (P-Ni-cloth) [89]. The conductive Ni cloths, which serve as electrodes, were produced by electroless plating of Ni on the PE cloths, whereas the P-Ni cloths were coated with parylene by a CVD process. The power cloth produced a power density of up to 393.7 mW m^−2^ at a frequency of 0.7 Hz and a load resistance of 70 MΩ. A wearable power unit was designed by integrating the TENG cloth and a flexible lithium ion battery (LIB) belt. After having been charged by the TENG cloth for three cycles, the LIB belt powered a heartbeat meter strap to remotely communicate with a smart phone, indicating the viability of the wearable smart electronics. Following that, an all solid-state flexible yarn supercapacitor and its integration with the TENG cloth for a self-charging power textile were demonstrated [90].

**Figure 7:**
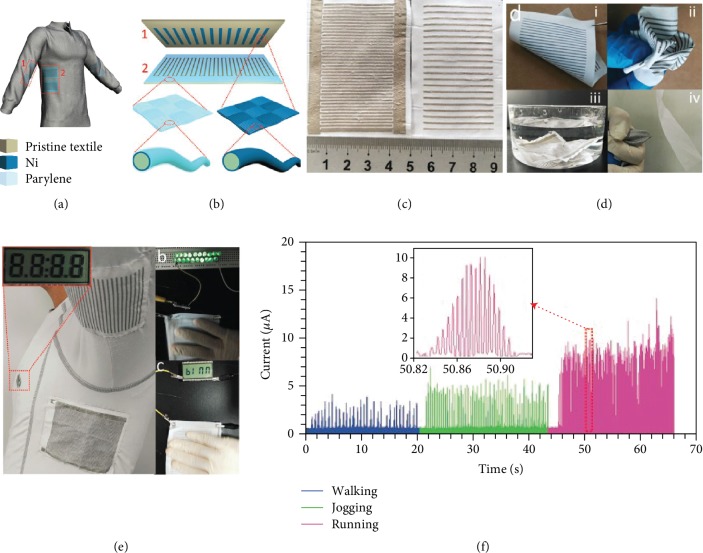
(a) The scheme of a power textile with a pair of TENG fabrics consisting of a slider fabric (1) in the sleeve and a stator fabric (2) underneath the arm. (b) The scheme of the configuration of the TENG fabrics. (c) A photo of a pair of TENG fabrics. (d) Photos showing the flexibility (i), softness (ii), washability (iii), and breathability (iv) of the TENG fabrics. (e) A photo of a power textile with a pair of TENG fabrics, which can light up a LCD panel (inset). (f) The rectified current of the TENG fabrics at different motion speeds of the human body. The inset shows the current of one sliding cycle of the TENG fabrics.

To make a textile-based energy-harvesting system, a grating-structured TENG fabric and its integration with a fiber-shaped dye-sensitized solar cell (FDSSC) were developed in 2016 [91]. As schemed in Figure 7(a), a power textile was designed with the sleeve fabric and the fabric underneath the arm to scavenge the swing energy of two arms during walking or running movements. The structure of a pair of TENG fabrics is depicted in Figure 7(b). To fabricate the TENG fabrics, a route of laser-scribing masking was developed. A polyester fabric sealed with commercial Kapton tape on both sides was put underneath a laser for drawing intended patterns as a mask for the subsequent ELD plating of Ni. After coating the exposed textile with a Ni film, the Kapton tape was then detached, yielding a conductive pattern of Ni film on one side of the fabric. Figure 7(c) is a pair of TENG fabrics derived from a white polyester cloth. The TENG fabric can be easily bent, wrapped, and immersed in water without damage, and possesses good breathability (Figure 7(d)). To demonstrate its application, a pair of TENG fabrics with 1 mm wide segments was knitted onto a common cloth as shown in Figure 7(e). A LCD screen can be easily lit up by swinging the slider fabric under the arm (see the inset). Meanwhile, the relative friction of the slider and stator fabric can light 20 green LED bulbs and a 5 × 2.5 cm LCD panel with characters of “binn.” The corresponding rectified current of the TENG fabrics at different speeds of human motion was given in Figure 7(f). Furthermore, TENG fabrics and FDSSCs were integrated together into a cloth as complementary power devices for harvesting both the energy of sunshine and human motions. The energy generated by the TENG fabrics and FDSSCs were stored in the battery for a stable power supply for wearable electronics.

Similarly, interdigitated conductive electrodes on a common textile were fabricated by laser-scribing masking and electroless deposition of conformal Ni coatings. Liu et al. reported large-area all-textile-based pressure-sensor arrays composed of a bottom interdigitated textile electrode and a top bridge of CNT-coated cotton fabric [92]. The textile sensor unit achieves high sensitivity (14.4 kPa^−1^), low detection limit (2 Pa), fast response (~24 ms), low power consumption (<6 *μ*W), and mechanical stability under harsh deformations, which is demonstrated to be able to recognize finger movements, hand gestures, acoustic vibrations, and a real-time pulse wave.

Jung et al. demonstrated a fabric-based wearable integrated energy device consisting of TENGs and SCs [93]. Electrical energy generated by a TENG was stored in a fabric-based SC with a symmetric structure of polyvinyl alcohol (PVA)/H_3_PO_4_ gel electrolyte sandwiched between two carbon fabric/CNT/RuO_2_ electrodes. A TENG was fabricated by arranging four complementary materials: PU and polyimide, PDMS, and Al. The TENG can harvest mechanical energy from vertical and horizontal motions between arms and the torso. A fabric-based TENG could generate output voltages and output currents up to 6 V and 55 nA, respectively, from rubbing movements, and about 15 V and 130 nA, respectively, from contact/release movements.

## 4. Conclusion and Prospection

The development of fabric-based TENGs is important for converting human motion energy into electricity and is relevant for the development of wearable electronics. The aforementioned discussion presents that fabric materials can offer a simple yet remarkably functional solution to design and fabricate low-cost TENGs for applications in sensors, heaters, and healthcare monitoring clothes. However, in many reported TENGs, the fabrics only serve as one or both substrates, as shown in Table 2. Namely, fabrics provide mechanical support and another conductive layer material is attached as the electrode. Because of this, their conductivity has not been shown to match that of metals. Low resistance is important for low power and highly portable applications. Increasing the thickness of the conductive layer can increase the conductance, but it also raises the concerns of comfortability and robustness. In addition, human motion is highly stochastic and irregular, which requires extra attention to the stability and robustness of device structural dynamics. To solve this issue, innovative structural and material designs, and novel packaging technologies need to be introduced into the fabrication of fabric-based TENGs. Furthermore, the time-average power output from wearable TENG systems has reached microwatts per square centimeter, but it is still several orders of magnitude away from the milliwatt power required for most real applications of microelectronic wearable systems. Hence, additional energy-harvesting and energy-storage units are required to fabricate self-powered systems with stable power output for various wearable and portable electronic devices. Last but not the least, to commercialize fabric-based TENGs as a wearable power source, important issues need to be addressed, including mass production, integration into clothes, biosafety, and less irritating material technology.

## Figures and Tables

**Table 1:** The output performance for fabric-based TENGs with different structures.

TENG	Voltage	Current	Peak power density	References
Nonwoven structure	500 V	20 *μ*A	153.8 mW/m^2^	[57]
	17 V	7 *μ*A	18 mW/m^2^	[60]
	22 V	70 *μ*A	46.5 mW/m^2^	[77]
	170 V	120 *μ*A	—	[78]
	368 V	78 *μ*A	33.6 mW/m^2^	[79]
	590 V	12.6 *μ*A	2.8 W/m^2^	[80]

Interdigital structure	2000 V	200 *μ*A	—	[81]
	120 V	20 *μ*A	3.2 W/m^2^	[91]
	15 V	130 nA	1.8 mW/m^2^	[93]

Weaving structure	40 V	210 *μ*A	—	[42]
	45 V	1.8 *μ*A	236.36 mW/m^2^	[44]
	24 V	1.5 *μ*A	12.5 *μ*W/m	[46]
	14.5 V	50 *μ*A	70 *μ*W/m^2^	[56]
	28 V	0.4 *μ*A	—	[58]
	90 V	1.2 *μ*A	—	[82]
	540 V	140 *μ*A	0.892 mW/m^2^	[83]
	1050 V	22 *μ*A	0.56 W/m^2^	[84]
	50 V	20 *μ*A	397.3 mW/m^2^	[89]
	30 V	18 *μ*A	—	[90]

Knitting structure	125 V	1.2 *μ*A	60 mW/m^2^	[43]

Sewing structure	2 V	200 nA	—	[45]

**Table 2:** Conductive substrates of fabric in triboelectric nanogenerators.

Conductive substrate	References
*CNT-coated fabric*: CNT-coated cotton thread, CNT stacked on PDMS, CNT cotton, and CNT-coated silk fiber	[41], [57], [59], [60], [92]
Carbon cotton	[93]
*Metal wire*: Al wire and stainless-steel wire	[42], [43], [44], [46]
*Metal foil*: Al foil, Cu foil, and Ni foil	[42], [45], [58], [80], [81], [83], [84]
*Commercial metal conductive fabric*: silver fabric, silver paste-coated PET fabric, Au textile, and Ni fabric	[45], [57], [78], [79], [82], [86], [88]
*Metal layer-coated fabric:* Ag nanowire-coated polyester fabric, Cu-coated PBT substrate, Ag coated on FEP film, Ag layer sputtered on nylon cloth, Ag nanowire-coated nylon cloth, Au-coated PET fabric, Au-coated polyurethane fabric, Cr/Au-coated nylon fabric, and Ni-coated polyester	[56], [58], [77], [80], [85], [87], [89], [90], [91], [92]
